# Artificial Intelligence in Genetics

**DOI:** 10.7759/cureus.52035

**Published:** 2024-01-10

**Authors:** Rohit S Vilhekar, Alka Rawekar

**Affiliations:** 1 Medical Genetics, Jawaharlal Nehru Medical College, Datta Meghe Institute of Higher Education and Research, Wardha, IND; 2 Physiology, Jawaharlal Nehru Medical College, Datta Meghe Institute of Higher Education and Research, Wardha, IND

**Keywords:** genetic disease, drug repurposing, artificial neural networks, deep learning, machine learning, artificial intelligence

## Abstract

The simulation of human intelligence in robots that are designed to think and learn like humans is known as artificial intelligence (AI). AI is creating a world that has never been seen before. By applying AI to do jobs that would otherwise take a long time, humans have the chance to improve our planet. AI has great potential in genetic engineering and gene therapy research. AI is a powerful tool for creating new hypotheses and helping with experimental techniques. From the previous data of a gene model, it can help in the detection of heredity and gene-related disorders. AI developments offer an excellent possibility for rational drug discovery and design, eventually impacting humanity. Drug development and discovery depend greatly on AI and machine learning (ML) technology. Genetics is not an exception to this trend, as ML and AI are expected to have an impact on nearly every aspect of the human experience. AI has significantly aided in the treatment of various biomedical conditions, including genetic disorders. In both basic and applied gene research, deep learning - a highly versatile branch of AI that enables autonomous feature extraction - is increasingly exploited. In this review, we cover a broad spectrum of current uses of AI in genetics. AI has enormous potential in the field of genetics, but its advancement in this area may be hampered in the future by a lack of knowledge about the accompanying difficulties that could mask any possible benefits for patients. This paper examines AI's potential significance in advancing precision genetic disease treatment, provides a peek at its use in genetic clinical care, examines a number of existing AI and ML uses in genetics, provides a clinician primer on critical aspects of these technologies, and makes predictions about AI's potential future applications in genetic illnesses.

## Introduction and background

The concept of creating robots is often considered the starting point for artificial intelligence (AI). Back in 1921, in his play "R. U. R" (Rossum's Universal Robots), writer Karel Capek introduced the term “robot,” which is derived from the Czech word “robota.” In the context of the play, it referred to a factory where bioengineered machines were used for labor under duress. Jumping forward to the middle of the 20th century, the term “robot” became immortalized in contemporary science fiction, thanks to Isaac Asimov's collection of short stories. Interestingly, even though the term was popularized relatively recently, the idea of humanoid automatons dates way back to the third century in China. The U.S. Department of Defense quickly grew interested in the numerous challenging mathematical problems that computers began to tackle in the following years. A new golden era then began with the use of logistic data mining and medical diagnosis following a period of slowdowns in the 1980s. Instruments with higher computational capacities were created. Today, AI is regarded as an area of engineering that employs fresh ideas and creative approaches to tackle complex problems. Computers may one day be as clever as people if advancements in technological speed, capacity, and software coding are made in the future. One cannot ignore the crucial role that modern cybernetics has played in the advancement of AI [1].

An AI system, sometimes known as an AI system, is a sophisticated piece of hardware or software that uses AI concepts to carry out activities that would typically need human intelligence. A machine learning (ML) system that was used to identify diabetic retinopathy in images of the retinal fundus received the first Food and Drug Administration (FDA) approval for an autonomous AI system in 2018 in a variety of medical sectors. Genetic engineering and AI have brought a new age of opportunities in biotechnology and customized medicine. AI contributes to predicting and optimizing genome editing methods such as CRISPR-Cas9. ML algorithms can analyze large-scale genetic sequence datasets, which can then be used to steer the development of more accurate and effective genome editing technologies by predicting probable off-target consequences [2]. Contrary to the previous generation of AI systems, which relied on the development of solid decision rules and the curation of medical information by specialists, more recent AI research has used ML techniques, which can take complicated interactions into consideration. Basic ML algorithms can be generally divided into supervised and unsupervised algorithms based on the types of tasks they are intended to accomplish. In order for supervised ML techniques to function, a large number of "training" instances must be gathered, each of which contains inputs (such as fundus images) and the required output labels (such as the presence or absence of diabetic retinopathy). The algorithm learns to create the appropriate output for a given input on new cases by examining the patterns in all of the labelled input-output pairs. The recent renaissance in AI has, to a large extent, been driven by the successful application of deep learning (DL), which involves training an artificial neural network (ANN) with many layers (that is, a 'deep' neural network) on huge datasets, to large sources of labelled data [3]. In genetics, AI refers to using sophisticated computational methods to analyze and interpret genetic data. This multidisciplinary discipline uses AI to decipher genetic data and provide academics and medical professionals with a better understanding of the complicated functions of the genome. By creating algorithms that best represent a set of data, ML focuses on the learning component of AI. ML employs subsets of data to produce algorithms that may use innovative or unconventional combinations of features and weights that cannot be deduced from first principles, in contrast to classical programming, in which an algorithm may be explicitly implemented using known features [4]. ML offers the potential to improve the accuracy and reliability of echocardiography, which is central to modern diagnosis and management of heart disease [5]. AI is thought to have human-like qualities displayed by machines. When a computer exhibits cognitive behavior similar to that of humans, such as learning or problem-solving, this phrase is employed [6]. The expanding scale and inherent complexity of biological data have encouraged a growing use of ML in biology to build informative and predictive models of the underlying biological processes. Precision medicine and "superhuman" powers are frequently linked to the rise of AI in medicine. At the same time, it is frequently forgotten that routine tasks make up a significant portion of a physician's day-to-day work and that assigning those tasks to AI would free up human workers' time for higher-value tasks that typically call for human qualities such as creativity, cognitive insight, meaning, or empathy [7]. AI in the field of computer science strives to replicate human reasoning, learning, and knowledge storage. Exciting possibilities exist for using medical imaging more effectively and efficiently, thanks to the potential new AI capabilities [8].

## Review

Methodology

**Figure 1:**
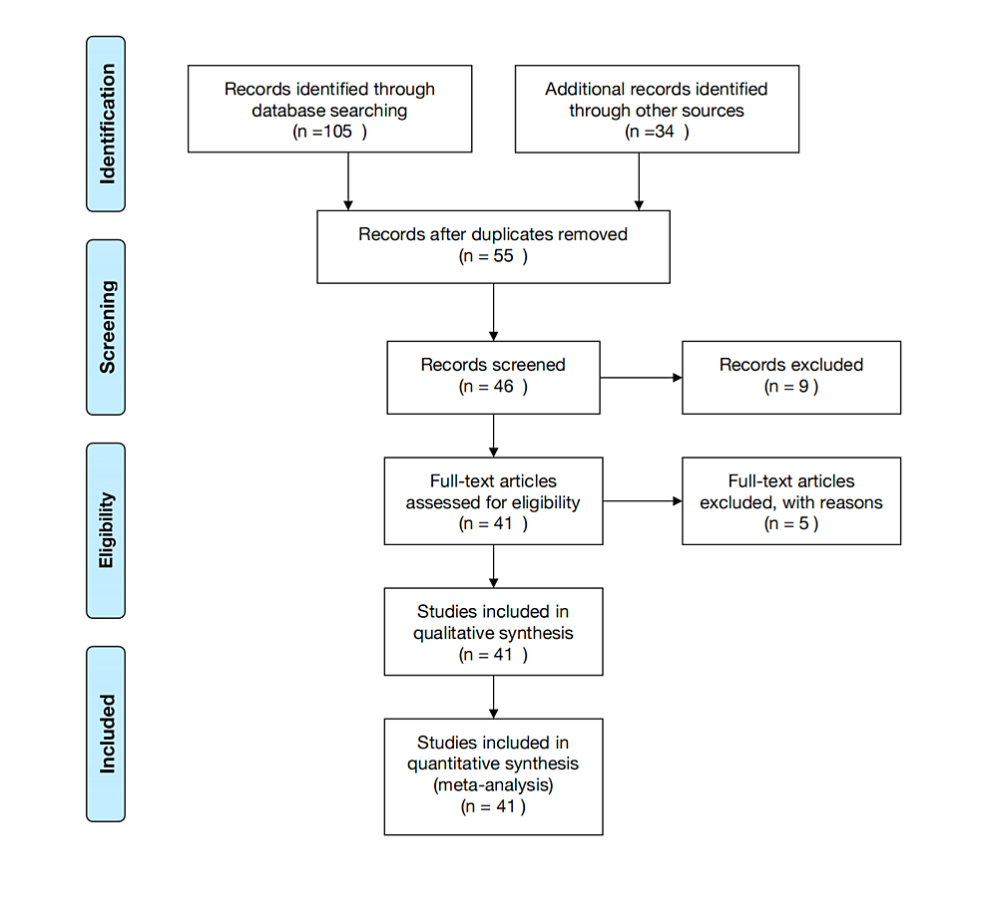
PRISMA flowchart of search strategy Adopted from the Preferred Reporting Items for Systematic Reviews and Meta-Analyses (PRISMA).

We looked up the Central Database and Medline using the Web of Science and PubMed, respectively. The keywords used in the search were “artificial intelligence,” “machine learning,” “deep learning,” “virtual screening,” “artificial neural networks,” “quantitative structure-activity relationship,” “drug repurposing,” “AI and ML”, “genetics disease,” and “genetics.” Reviewing the papers' references, we also sought further studies. These computerized searches identified papers, and the bibliographies of those studies were reviewed for relevant citations (Figure 1).

Genome sequencing by artificial intelligence

AI has dramatically improved the process of genome sequencing, which is figuring out the nucleotide order in a person's deoxyribonucleic acid (DNA). This is how AI aids in the sequencing of genomes. Because the majority of hospitals have begun to incorporate medical electronic records into their patient care operations, healthcare data are now more easily accessible through computers than through any other source [9]. The patterns of patient health trajectories can be learned by an ML algorithm. Using information that goes well beyond the particular doctor's practice experience, this facility can assist doctors in accurately anticipating future events [10]. Working from crystallographic data collected by Rosalind Franklin and Maurice Wilkins, Watson and Crick famously determined the three-dimensional structure of DNA in 1953. This work contributed to a conceptual framework for both DNA replication and encoding proteins in nucleic acids. However, it took some time before scientists were able to "read" or sequence DNA. Strategies used to determine the sequence of protein chains did not appear to be easily transferable to nucleic acid studies because DNA strands were much longer and comprised fewer, more comparable units than protein molecules. It was necessary to establish new strategies [6]. More than 1,800 gene therapy clinical trials have been approved globally, either currently ongoing or completed. The most frequently employed gene transfer vehicles in clinical studies have been adenoviral vectors, retroviral vectors, and bare plasmids [11]. Ribonucleic acid sequencing (RNA-Seq), a recently developed transcriptome profiling technique, uses deep-sequencing technology. Studying eukaryotic transcriptomes with this technology has already led to changes in our knowledge of their size and complexity. Additionally, RNA-Seq provides a significantly more precise way to determine the quantities of transcripts and their isoforms than alternative methods. he RNA-Seq method, along with its real-world applications and the progress achieved in defining different eukaryotic transcriptomes to date, is a promising method and is changing the way we think about gene expression, revealing complex biological processes and offering insightful information about a wide range of subjects such as environmental science, agriculture, and medicine [12]. Retrospective studies suggest that more complex and precise prognostic models can be built with raw data from medical imaging. Large integrated health systems have already used simple ML models to automatically identify hospitalized patients who are at risk for transfer to the intensive care unit [10]. For a long time, computational tools have been crucial to drug discovery and design, which has changed the entire drug design process. Traditional computational approaches still have a lot of drawbacks, such as time costs, computational costs, and reliability. All of these computational drug design barriers could be eliminated by AI, and, as a result, computational methods could play a bigger part in drug development [13]. The ML strategy includes cross-validation, feature selection using the information gain method, merging three separate algorithms, and a majority vote for the final scoring [14]. Some applications of AI genome sequencing are mentioned in Table 1.

**Table 1:** AI-powered genome sequencing aspects AI, artificial intelligence

Aspect	Description
1. Accelerated sequencing	AI shortens the time and expense associated with genome sequencing.
2. Error reduction	AI decreases errors, increasing the accuracy of genome sequencing.
3. Variant identification	AI swiftly and correctly pinpoints genetic variations related to diseases or traits.
4. Personalized medicine	AI uses genomic data analysis to personalize medicines based on each patient's genetics.
5. Population studies	Large-scale datasets are analyzed using AI to provide insights about population-level genetic variants.
6. Structural variation analysis	Large-scale genomic rearrangements and structural changes can be found with the help of AI.
7. Data integration	AI combines clinical, environmental, lifestyle, and genomic data to provide thorough insights.
8. Scalability	AI makes it possible to scale up sequencing operations and handle enormous genomic datasets.
9. Ethical considerations	Sensitive genomic data storage and dissemination provide ethical difficulties.
10. Regulatory compliance	AI ensures that AI-driven sequencing complies with regulatory requirements and protects user data.

Utilizing the drug design approach, this technology can be applied to the progress of genetics. We can make significant gains if we put in more labor and time. The high-throughput next-generation sequencing (HT-NGS) techniques were chosen as the 2007 method of the year because they offer new possibilities and have a significant impact on mammalian genomics research. The route to gaining acceptability for these revolutionary technologies, however, was not a straightforward one. The initial step of the HT-NGS technique was using a sensitive charge-coupled device (CCD) camera to find the following fluorescently tagged base (reversible terminator) in the lengthening DNA chain. This was carried out simultaneously on a large number of DNA samples on DNA chips that were linked to either beads or a planar substrate, therefore reducing reaction volumes in a miniature microsystem. The dye was removed, and the terminator was changed into a regular nucleotide in the following step. To determine the following base in the sequence, this cycle and technique were repeated. The concept outlined in this application is somewhat similar to that employed in the so-called next-generation devices currently being marketed by firms like Roche, Illumina-Solexa, Application binary interface (ABI), Helicos, and others [6].

Proteomics is a new category of "omics" that has grown quickly, particularly in the pharmaceutical industry. Marc Wilkins coined the term “proteome” in 1995. The study of proteins' relationships, biological functions, makeup, and architectures is known as proteomics. In comparison to genomics, proteomics offers a greater grasp of the composition and operation of the organism [15]. In other treatment fields, neurological illnesses predominate by a wide margin over diseases. However, due to lengthy timetables and high attrition rates, discovering medications for illnesses of the central nervous system (CNS) continues to be the most difficult area of drug development. AI and ML have emerged as vital tools to derive relevant insights and enhance decision-making in drug development as a result of the enormous growth of biomedical data made possible by cutting-edge experimental methods [16]. Any organism's ability to develop and survive depends on its essential genes. To reduce the amount of resources needed for essentiality assays, the ML strategy is a supplement to the experimental approaches. Previous research has shown that in order to improve prediction, it is necessary to build a solid gold standard that serves as the class label for the train data. This will improve the generalizability of prediction models across species. Findings also indicate that detecting conditionally essential genes is a serious weakness of the ML approach [17]. Overall, the proteomics workflow has not changed much over the past 20 years despite orders of magnitude increases in data resolution, accuracy, sensitivity, and performance. Proteins are broken down by enzymes into peptides, which are then separated by chromatography, ionized by electrospray (ES), and mass-analyzed before being fragmented. The experiment's main goal is to detect and measure as many of the eluting peptides as we can. Additionally, data completeness is crucial, particularly for ML applications. Numerous acquisition strategies are mentioned in Table 2, each with a particular trade-off between speed, resilience, quantitative correctness, and the depth of proteome coverage (percent of the expressed proteome collected). Peptides from various experimental states are isotopically encoded using chemical labelling techniques, and the patterns of "reporter ions" are used to quantify the differences between them. Proteomics is a multi-step process that requires meticulous control at each stage to prevent non-biological influences from interfering with protein expression and interaction. Laboratory tests can be used to study cells, DNA, or tiny molecules, but proteins are the class with the greatest number and frequency of assays, which reflects their crucial importance in medicine. These facts also point to the enormous medical potential of novel protein-based biomarkers. Furthermore, enzymatic tests or immunoassays are frequently used in clinical assays to examine a particular target. The ability to quantify several proteins simultaneously and with considerably higher specificity is one promise of mass spectrometry (MS)-based proteomics. However, because of conceptual and technological constraints, this promise is just now beginning to be fulfilled [18]. Different acquisition methodologies are used in the field of AI genome sequencing to improve capabilities, increase accuracy, and expand the applications of genomic data analysis given in Table 2.

**Table 2:** Some acquisition strategies along with brief descriptions AI, artificial intelligence

Acquisition strategies	Description
Partnerships and collaborations	Access to large and varied genetic datasets can be gained through forming alliances and working together with academic institutions, biotechnology businesses, and healthcare organizations. These kinds of partnerships make it easier to develop and validate AI models more broadly.
Mergers and acquisitions	Businesses in the AI and genomics industries may merge or buy other companies to pool resources and expertise. Fusing AI technology with already existing genome sequencing tools can spur innovation and produce all-encompassing solutions.
Data licensing and sharing	Large-scale genomic dataset acquisition and curation are areas of expertise for some organizations. By granting licenses or sharing these datasets with AI-focused businesses, strong machine learning models can be developed and trained, improving the precision and applicability of genetic investigations.
In-house data generation	Businesses that leverage AI to drive genomic sequencing may make investments in internal data production resources. This gives more control over the caliber and variety of the information used to train AI models since it entails directly gathering, processing, and analyzing genomic data.
Strategic alliances with sequencing platforms	AI-focused businesses are able to directly incorporate their algorithms into the sequencing workflow by forming strategic agreements with DNA sequencing platform suppliers. This partnership makes it possible to analyze and understand data in real time, which expedites the process of sequencing a genome.
Crowdsourcing and citizen science initiatives	Including the public in citizen science or crowdsourcing projects can be a useful acquisition method. Encouragement of individuals to share their genomic data for study enables AI-powered platforms to access a larger and more varied pool of genetic data.
Investment in research and development	By allocating resources toward internal research and development, organizations can foster innovation in AI algorithms tailored for the interpretation of genomic data. This tactic aids in the development of proprietary technologies and keeps one ahead of the curve in the field of genomics and AI convergence.
Open-source collaboration	Collaboration and information sharing are encouraged by using or contributing to open-source initiatives in the AI and genomics sectors. Through pooled knowledge, open-source projects offer a platform for the creation and enhancement of AI algorithms for genome sequencing.
Clinical trial collaborations	Clinical genetic data can be accessed through working with pharmaceutical companies and clinical trial activities. Real-world patient data can be used to enhance and test AI applications in genome sequencing, leading to more accurate and therapeutically meaningful outcomes.
Global expansion and market access	AI-powered genome sequencing firms are able to collect a vast array of genetic variations by expanding their operations worldwide and acquiring access to varied populations. This tactic improves the AI models' resilience and generalizability to various racial and geographic groups.

Precision making in genome

Our world is experiencing a technological revolution that is being fueled by ever-increasing computational capacity. High-throughput computation, high-throughput genomics, and “big data” resources from biobanking have grown in significance for genetics research. Recently, the use of precision medicine has gained much support. In order to maximize individual therapy, it centers on the unique patient, taking into consideration genetic, biomarker, phenotypic, or psychological aspects [19]. The Online Mendelian Inheritance of Man estimates that roughly 4000 genes have mutations that can cause phenotypes [20].

The last 10 years have seen a significant increase in investment in techniques to promote precision medicine, leading to new treatments, increased knowledge of disease mechanisms, and, ultimately, disease prevention. Precision medicine emphasizes finding the best strategies and individualized care based on a person's genetic, environmental, and lifestyle characteristics. The results of the Human Functional Genomics Project (HFGP), which focused on 500 healthy adult individuals, provide indisputable proof of human biological variety in both health and disease. This has been well demonstrated by numerous studies using immune cells (cytokines) as an endpoint, demonstrating that cytokine types and amounts rely on environmental factors (such as the time of year), genetic background, and intestinal microbiota composition. In addition, the most recent research from the HFGP revealed that 11 distinct host variables jointly accounted for up to 67% of inter-individual variation in the production of activated cytokines in healthy people [21]. With patient-level AUROCs (The AUROC is calculated as the area under the ROC curve) of 0.85, 0.75, 0.74, 0.79, 0.81, and 0.67 on the held-out dataset (i.e., the test dataset), Coudray et al. developed a DL-based image analysis method for mutation prediction in non-small lung cancer in their seminal study, which was published in 2018. This method was used to predict mutations in serine/threonine kinase 11 (STK11), epidermal growth factor receptor (EGFR), FAT1, SETBP1, and KRAS (Kirsten rat sarcoma virus) [22].

The ANN model uses the “relu” activation function and consists of three hidden layers, each with 64 neurons. The “sigmoid” activation is used by the output layer to perform binary classification. In total, 300 decision trees are used to generate the random forest model. The training data are used to train both models, and the test data are used to evaluate them. In real life, you would substitute your own data for the dataset loading portion and modify the architecture and hyperparameters in accordance with the particulars of your issue. For a more thorough analysis, you might also think about utilizing cross-validation. Synthetic gene circuits allow programming in DNA the expression of a phenotype at a given environmental condition. The recent integration of memory systems with gene circuits opens the door to their adaptation to new conditions and their re-programming [23]. The creation of algorithms that can extrapolate a set of rules from a specified “training” dataset is one of the main objectives of ML. In what is known as "supervised learning," the algorithm should ideally be able to correctly categorize previously unseen datasets into the proper categories. One method of this categorization, also known as sorting, involves categorizing all data inputs into one of two states, such as being above or below a specific linear threshold. The term "linear classification" refers to this kind of supervised learning, and numerous algorithms have been created to accomplish this goal [24]. ML models that were used to identify patients with positive outcomes were trained using all of the parameters. For the deep neural network model, there were three hidden layers with a total of 15 ANNs each. For the random forest model, 300 decision trees were used. To evaluate the accuracy of the ML models, we generated the ASTRAL (Accurate Species TRee ALgorithm) score, one of the well-known prognostic scoring systems for acute stroke. In simple words, it means the utilization of deep neural networks and random forest models for predicting positive outcomes in acute stroke patients. The models were trained using a comprehensive set of parameters, and their accuracy was evaluated using the ASTRAL score. The use of ML in this context aims to enhance the prediction and understanding of patient outcomes based on various input factors [25]. The copy number (CN) itself, which is a simple series of chemical processes, will be the first (basic) version. Additionally, because it solely consists of micro-reversible reactions with mass-action kinetics, it is thermodynamically consistent. Although small, this first version makes a lot of enzymatic multiplicity assumptions that are unlikely to be true. As a result, we will suggest a second iteration of the model that is biologically plausible in the sense that it may be expressed in terms of well-known biochemical motifs but is not thermodynamically explicit. This system and the previous one vary primarily in that the former is segmented. This divided system will henceforth be referred to as c-CN. DNA strand displacement (DSD), a sort of DNA-based computing, is used to develop the d-CN, a variant of the CN. DSD is a biocompatible molecular computing paradigm that is fully based on how DNA strands interact and Watson-Crick complementarity. By this, we mean that DSD computers have the potential to be utilized to regulate molecular systems because they may theoretically be injected into animals and interact with their biochemistry. It has been demonstrated that DSD systems are capable of doing any type of computing, including the emulation of any chemical process network. DSD systems are reasonably simple to materialize experimentally, and their behavior can also be precisely anticipated using simulation tools like Visual DSD or Peppercorn. A wide range of computational techniques and resources are now available for creating DNA-based circuits. Multiple initiatives to create intelligent DSD systems have been made. Examples include oscillators, switches, logic gates, linear-threshold circuits, and consensus procedures [26]. Microarrays, particularly the Illumina HumanMethylation Infinium BeadArray, are one of the most widely used techniques for determining the methylation profiles over the entire genome [27]. The natural selection theory is the foundation of the genetic algorithms, which are crucial in solving such complex issues. Numerous problems are optimized in the literature using genetic algorithms. These techniques have given computational biologists efficient ways to locate the ideal values for huge datasets. Image reconstruction has been done using genetic algorithms. These algorithms build on sub-algorithms to increase their precision and accuracy [28].

Thus, genetic biomarkers have particular promise for psychiatric illnesses. Over the past 10 years, genome-wide association studies of prevalent diseases have become more sophisticated, building the information foundation for more accurate genetic risk prediction at the individual level. In this study, we cover the underlying ideas behind assessing genetic risk using modern techniques, the advantages and disadvantages of various strategies, utility evaluations, and applications for various psychiatric diseases and associated features [29]. Utilizing sizable multidimensional biological datasets that contain individual heterogeneity in genes, function, and environment, precision medicine aims to build and optimize the pathway for diagnosis, therapeutic intervention, and prognosis. This gives doctors the chance to individually adapt early interventions, whether they are therapeutic or preventative in nature. AI systems may now reasonably forecast risk for several malignancies and cardiovascular diseases from existing multidimensional clinical and biological data by utilizing high-performance computer capabilities [30].

Future outlook and obstacles

The science of AI, which is rapidly expanding, has applications to genetic diseases that have the potential to revolutionize how many chronic conditions are diagnosed and treated. Algorithms supporting predictive models for the risk of developing genetic disorders or their complications have been built using ML principles [31]. Although preimplantation genetic testing for aneuploidy and time-lapse incubators have been developed to help raise the likelihood of a live birth, the results are still far from ideal. AI is rapidly being used in the medical industry to help increase the success rates of in vitro fertilization (IVF) procedures [32]. Large and complicated genomic datasets are processed using a particular form of AI algorithm, known as DL, in various fields, such as clinical genomics [33]. It is now possible to address unmet clinical needs in genetics and uncover novel mechanistic insights, thanks to the large datasets that have quickly accumulated from electronic medical records, high-definition multi-omics (including genomics, proteomics, transcriptomics, and metagenomics), and imaging modalities (endoscopy and endomicroscopy). Although the use of AI methods has made it simpler to analyze, combine, and interpret huge genetics datasets, the requirement for objective prospective validation studies, as well as the substantial heterogeneity in AI methods, datasets, and clinical outcomes, are currently preventing the use of AI in clinical practice [34]. The field of heart failure (HF) has benefited from enhanced biomarker discovery, thanks to technological advancements. Using high-throughput omics systems to profile HF at the level of genes, transcripts, proteins, and metabolites has improved the efficiency of a traditionally long and arduous process. Additionally, advances in AI have simplified the understanding of big omics datasets and enhanced analysis. Clinicians can benefit from the use of omics and AI in biomarker discovery by discovering signs of HF risk, monitoring care, figuring out prognoses, and creating druggable targets. AI has the potential to enhance HF patient care when used together [35]. Patient care may advance with the development of AI and ML technologies. Applications, as mentioned in Table 3, include cancer diagnosis and monitoring, identifying at-risk populations of people, classifying genetic variations, and even predicting the ancestry of a patient. This article discusses the difficulties and factors to be taken into account when implementing these tools in clinical practice, as well as some recent and potential applications of AI in genetic medicine [36]. The scientific community is growing more interested in developing the current therapeutic approaches to treating cancer, even if surgery, chemotherapy, and radiotherapy will continue to be the gold standard for cancer treatment for many years to come. In the future, the use of computational input and support will produce a real-world clinical environment, and a significant technological revolution will avoid emotional issues, cultural and moral norms, and exhaustion in the real-time prediction and diagnosis of human health-related disorders [37]. The applications listed in Table 3 demonstrate the various and significant applications of genetics and AI in the fields of genetics and healthcare. Examples include cancer detection and monitoring, identifying at-risk populations, classifying genetic variants, and predicting patient ancestry.

**Table 3:** Cancer diagnosis and monitoring, identifying at-risk populations, classifying genetic variations, and predicting patient ancestry are examples of the diverse and impactful applications of genetics and AI in healthcare and genetics AI, artificial intelligence

Applications	How AI is applied	Impact
Cancer diagnosis and monitoring	Genomic data can be analyzed by machine learning models to find patterns linked to cancer. These models can help in cancer recurrence probability prediction, subtype categorization, and early diagnosis.	AI-assisted early diagnosis and monitoring lead to more individualized and efficient treatment plans, which enhance patient outcomes.
Identifying at-risk populations	Large-scale genetic databases can be analyzed by AI algorithms to determine which people are more susceptible to a given disease, such as inherited disorders or complicated disease susceptibility.	Public health initiatives can be strengthened by implementing screening programs, preventive measures, and targeted interventions for populations that are at risk.
Classifying genetic variations	Genetic variants can be categorized and interpreted by machine learning algorithms, which can differentiate between potentially hazardous and benign mutations. Understanding the genetic foundation of diseases requires knowledge of this.	Precise categorization of genetic variants facilitates the diagnosis of hereditary illnesses, directs therapeutic choices, and expands our comprehension of the genetic foundations of ailments.
Predicting ancestry of a patient	AI systems are able to predict an individual's ancestral ancestry by analyzing genetic markers. To do this, the genetic profile is compared to reference datasets made up of various demographic groups.	Because various genetic variants and susceptibilities might be associated with particular populations, ancestry prediction holds potential implications in personalized medicine. It also helps with customized healthcare planning.

## Conclusions

This review's objective is to outline the current course of human genetic research in light of developments in phenome-wide research, a cutting-edge area of study that is frequently contrasted with genome-wide research. The previous patterns in human genetic research should always be evaluated before discussing potential future trajectories. Genomic data interpretation has been sped up by AI-driven techniques, allowing for more accurate diagnosis and personalized treatment regimens for people with genetic illnesses. New opportunities for early intervention and prevention have been made possible by the capacity to anticipate illness risk and consequences based on genetic information. Ensuring equal access to these technologies across a varied population and addressing ethical issues about privacy and data security are some of the challenges presented by the integration of AI in genetics. Collaboration between geneticists, physicians, and AI experts will be essential to leveraging the advantages of AI in genetics as the field develops. AI-powered genetics has the potential to change healthcare by delivering more focused, effective, and tailored methods for illness management and prevention, provided that ethical, legal, and social ramifications are carefully considered.
